# Quality in computed tomography angiography: we start with what can be considered normal

**DOI:** 10.1590/0100-3984.2022.55.4e2-en

**Published:** 2022

**Authors:** Marcelo Souto Nacif

**Affiliations:** 1 Adjunct Professor, Department of Radiology, Universidade Federal Fluminense (UFF), Niterói, RJ, Brazil. Email: msnacif@gmail.com.

The technique for acquiring any type of image that requires knowledge of fluid dynamics and contrast transit times through the various vessels of the body has a direct impact on the quality of the vascular contrast enhancement and can hinder the interpretation of examinations. Therefore, knowledge of the mean transit time of intravenous contrast media and of all of the factors that may confound its interpretation is extremely necessary. Those who were practicing radiologists 20 or more years ago and who, at that time, needed to carry out emergency examinations to detect pulmonary thromboembolism or aortic dissection in first-generation helical scanners know that the concept of the mean time of arrival of the contrast directly implies the end result of the examination.

In the previous issue of Radiologia Brasileira, Borges et al.^(1)^ retrospectively evaluated the pulmonary vascular circulation time in patients ≥ 18 years of age with normal cardiac volume and function, no history of heart failure, and no known valvular or lung disease, who underwent coronary computed tomography angiography with contrast medium infusion at 4 mL/s. A total of 43 patients were included in the study. The authors found that the circulation time from the pulmonary trunk to the ascending aorta correlated significantly with the left ventricular ejection fraction (r = -0.36; *p* = 0.015) and with the cardiac index (r = -0.29; *p* = 0.05).

In the Borges et al.^(1)^ study, the mean transit time of the contrast medium was 3.0 ± 2.4 s from the peripheral injection site to the superior vena cava and 2.9 ± 1.4 s from the superior vena cava to the pulmonary trunk. The mean transit time from the pulmonary trunk to the ascending aorta was 7.2 ± 2.2 s. Therefore, the total time elapsed between the peripheral injection of the contrast agent and its arrival in the ascending aorta was 13.0 ± 2.56 s^(1)^.

To deepen the discussion, it is relevant to consider that the mean height of the individuals in the population studied by Borges et al.^(1)^ was 163.7 ± 7.3 cm; that is, with a standard deviation ranging from 156.4 cm to 171.0 cm. The mean interval of the standard deviations for the circulation time between the pulmonary trunk and the ascending aorta was 4.4 s, and the mean transit time was 7.2 ± 2.2 s^(1)^. In view of that, some points need to be discussed more broadly and in greater depth so that we do not misinterpret the results of the study.

When performing any examination involving the use of an injection pump, it is essential to talk to patients before the examination. Instructing patients in how to perform a breath-hold during image acquisition should be encouraged at every facility. The main technical error made in chest examinations is an inspiratory breath-hold with a large amount of air inside the lungs, which results in a significant increase in intrathoracic pressure and a Valsalva-like effect. When that happens, even if an injection pump with a high infusion rate is being used, it is common to see a large amount of contrast in the subclavian artery and reflux of the contrast through the intercostal or paravertebral veins. That will have a direct effect on the contrast transit time.

One factor that cannot be overlooked is the viscosity and temperature of the intravenous contrast medium. Intravenous contrast media need to be warmed to body temperature to reduce viscosity, thus allowing greater injection velocity^(2)^.

Thoracic outlet syndrome is a clinical condition that can cause confusion during dynamic contrast-enhanced studies of the chest. In most cases, such examinations are performed with the arm of the patient extended alongside the head. When that position is assumed, any compression in the passage of the subclavian vein between the first rib and the anterior scalene muscle or any variation in the insertion of the pectoralis minor muscle can obstruct the transit of the contrast medium. In such cases, the examination should be performed with the arms down, alongside the body^(3)^. The contrast medium infusion rate will also change the mean contrast transit times. Therefore, the mean times after injection of a contrast medium at 3 mL/s will differ from those observed after injection of the same contrast medium at 5.5 mL/s.

Another important factor is the size of the patient. For example, the distance for the contrast medium to travel will be greater in tall, long-armed basketball players than in Olympic gymnasts, who typically have a smaller stature, shorter arms, and a smaller vena cava. That also influences the size of the pulmonary vascular bed and the contrast transit time to the ascending aorta. In the population studied by Borges et al.^(1)^, with a mean standard deviation interval of 14.6 cm, the mean transit time in the standard deviation interval was 4.4 s.

There are some interesting data in the study conducted by Skrok et al.^(4)^, who injected the contrast medium at a rate of 5 mL/s and reported a mean transit time of 6.4 s. In the Borges et al. study^(1)^, the mean transit time was 7.2 s when the contrast medium was injected at a rate of 4 mL/s, which shows the importance of the infusion rate.

There are various factors that can alter the circulation time. Such factors include venous obstruction (due to thrombosis of the subclavian vein, brachiocephalic trunk, or superior vena cava in patients on chemotherapy), thoracic outlet syndrome, and any type of mediastinal mass that compresses the superior vena cava, as well as intracardiac shunts and ventricular or valve dysfunction.

Although there is still a scientific argument for expanding this type of study, the knowledge provided by Borges et al.^(1)^ helps improve numerous protocols, as well as serving as a basis for education and guidance for technicians and biomedical personnel. In this context, given the relevant role of radiologists in training and assessing the quality of protocols for computed tomography angiography, the article authored by Borges et al.^(1)^ merits special attention, because knowledge of the patterns of normality have an impact on medical practice.
